# Comparative analysis and assessment of *M. tuberculosis* H37Rv protein-protein interaction datasets

**DOI:** 10.1186/1471-2164-12-S3-S20

**Published:** 2011-11-30

**Authors:** Hufeng Zhou, Limsoon Wong

**Affiliations:** 1 NUS Graduate School for Integrative Sciences & Engineering, National University of Singapore, 28 Medical Drive, Singapore 117456; 2 School of Computing, National University of Singapore, 13 Computing Drive, Singapore 117417

## Abstract

**Background:**

*M. tuberculosis* is a formidable bacterial pathogen. There is thus an increasing demand on understanding the function and relationship of proteins in various strains of *M. tuberculosis*. Protein-protein interactions (PPIs) data are crucial for this kind of knowledge. However, the quality of the main available *M. tuberculosis* PPI datasets is unclear. This hampers the effectiveness of research works that rely on these PPI datasets. Here, we analyze the two main available *M. tuberculosis* H37Rv PPI datasets. The first dataset is the high-throughput B2H PPI dataset from Wang et al’s recent paper in *Journal of Proteome Research*. The second dataset is from STRING database, version 8.3, comprising entirely of H37Rv PPIs predicted using various methods. We find that these two datasets have a surprisingly low level of agreement. We postulate the following causes for this low level of agreement: (i) the H37Rv B2H PPI dataset is of low quality; (ii) the H37Rv STRING PPI dataset is of low quality; and/or (iii) the H37Rv STRING PPIs are predictions of other forms of functional associations rather than direct physical interactions.

**Results:**

To test the quality of these two datasets, we evaluate them based on correlated gene expression profiles, coherent informative GO term annotations, and conservation in other organisms. We observe a significantly greater portion of PPIs in the H37Rv STRING PPI dataset (with score ≥ 770) having correlated gene expression profiles and coherent informative GO term annotations in both interaction partners than that in the H37Rv B2H PPI dataset. Predicted H37Rv interologs derived from non-*M. tuberculosis* experimental PPIs are much more similar to the H37Rv STRING functional associations dataset (with score ≥ 770) than the H37Rv B2H PPI dataset. H37Rv predicted physical interologs from IntAct also show extremely low similarity with the H37Rv B2H PPI dataset; and this similarity level is much lower than that between the *S. aureus* MRSA252 predicted physical interologs from IntAct and *S. aureus* MRSA252 pull-down PPIs. Comparative analysis with several representative two-hybrid PPI datasets in other species further confirms that the H37Rv B2H PPI dataset is of low quality. Next, to test the possibility that the H37Rv STRING PPIs are not purely direct physical interactions, we compare *M. tuberculosis* H37Rv protein pairs that catalyze adjacent steps in enzymatic reactions to B2H PPIs and predicted PPIs in STRING, which shows it has much lower similarities with the B2H PPIs than with STRING PPIs. This result strongly suggests that the H37Rv STRING PPIs more likely correspond to indirect relationships between protein pairs than to B2H PPIs. For more precise support, we turn to *S. cerevisiae* for its comprehensively studied interactome. We compare *S. cerevisiae* predicted PPIs in STRING to three independent protein relationship datasets which respectively comprise PPIs reported in Y2H assays, protein pairs reported to be in the same protein complexes, and protein pairs that catalyze successive reaction steps in enzymatic reactions. Our analysis reveals that *S. cerevisiae* predicted STRING PPIs have much higher similarity to the latter two types of protein pairs than to two-hybrid PPIs. As H37Rv STRING PPIs are predicted using similar methods as *S. cerevisiae* predicted STRING PPIs, this suggests that these H37Rv STRING PPIs are more likely to correspond to the latter two types of protein pairs rather than to two-hybrid PPIs as well.

**Conclusions:**

The H37Rv B2H PPI dataset has low quality. It should not be used as the gold standard to assess the quality of other (possibly predicted) H37Rv PPI datasets. The H37Rv STRING PPI dataset also has low quality; nevertheless, a subset consisting of STRING PPIs with score ≥770 has satisfactory quality. However, these STRING “PPIs” should be interpreted as functional associations, which include a substantial portion of indirect protein interactions, rather than direct physical interactions. These two factors cause the strikingly low similarity between these two main H37Rv PPI datasets. The results and conclusions from this comparative analysis provide valuable guidance in using these *M. tuberculosis* H37Rv PPI datasets in subsequent studies for a wide range of purposes.

## Background

Each year millions of tuberculosis patients perish, and fully one-third of the world’s population is infected with the causative agent of this disease, *M. tuberculosis*[1]. *M. tuberculosis* H37Rv is one of first fully sequenced *M. tuberculosis* strains [2]. With the increasingly intensive research focused on this pathogen around the world, *M. tuberculosis* H37Rv protein-protein interaction (PPI) data has become an important source of protein function and relationship information for relevant studies in microbiology, molecular biology, computational biology and medicine. However, *M. tuberculosis* H37Rv PPI data is far from complete and accurate. Hitherto predicted *M. tuberculosis* H37Rv PPIs in the STRING database (version 8.3 contains 248,574 PPIs covering 3,965 proteins in H37Rv) [3] have seen the most frequent used [4-6] because large-scale experimental PPI datasets have not been available until recently. The first large-scale proteome-wide PPI dataset of H37Rv was produced in 2010 using a high-throughput bacterial two-hybrid (B2H) approach [7]; it comprises 8,042 PPIs covering 2,907 proteins. No doubt in the foreseeable future, increasingly more studies on *M. tuberculosis* will be based on both of these datasets.

There is an extremely low overlap of just 276 protein-protein interactions shared between the 8,042 H37Rv PPIs in the B2H dataset and the 248,574 predicted H37Rv PPIs in STRING. It is the objective of this work to investigate the cause of this unexpectedly low overlap. We hypothesize that this low overlap between the two datasets may be due to (i) the B2H dataset is poor in quality, (ii) the STRING dataset is poor in quality, and/or (iii) the STRING dataset does not correspond to direct physical protein-protein interactions.

In order to test the quality of these two *M. tuberculosis* H37Rv PPI datasets, we evaluate them based on correlated gene expression profiles, coherent informative GO term annotations, and conservation in other organisms. Two proteins that interact are expected to be expressed at the same time and space; thus their underlying genes are likely to exhibit correlated expression profiles. Two proteins interact to effect a biological process or molecular function; thus they are expected to be annotated to some GO terms in common or GO terms that are closely related. Many protein-protein interactions are expected to be conserved across several organisms that have common ancestry; thus real protein interactions are likely to coincide with interologs from such organisms.

These assessments indicate that H37Rv B2H PPIs agree less well with correlated gene expression profiles, coherent informative GO term annotations, and conservation in other organisms than H37Rv STRING PPIs (with score ≥ 770) . This suggests that PPIs in the H37Rv B2H dataset may contain a high level of noise (false positives).

*S. aureus* is a bacterial pathogen with available high-throughput experimental physical PPI data; and it is close to *M. tuberculosis*. Thus interologs conserved in *S. aureus* should be very likely conserved in *M. tuberculosis*. However, H37Rv predicted physical interologs from IntAct show quite low similarity with the H37Rv B2H PPI dataset; and this similarity level is lower than that between the *S. aureus* MRSA252 predicted physical interologs from IntAct and *S. aureus* MRSA252 pull-down PPIs. This suggests the H37Rv B2H dataset may also be missing many real PPIs (false negatives).

To further confirm the suspected low quality of the H37Rv B2H PPI dataset, we compare this *M. tuberculosis* H37Rv B2H dataset with representative two-hybrid PPI datasets in other species (*C. jejuni*, *Synechocystis* and *S. cerevisiae*) [8-10]. These comparative analyses demonstrate that the quality of the *M. tuberculosis* H37Rv B2H PPI dataset is poorer than other two-hybrid PPI datasets.

As mentioned earlier, the H37Rv STRING PPI dataset (with score ≥ 770) , comprising entirely of PPIs predicted using a variety of methods, shows good agreement with correlated gene expression profiles, coherent informative GO term annotations, and conservation in other organisms. However, protein pairs that are functionally linked are also expected to agree well with correlated gene expression profiles, coherent informative GO term annotations, and conservation in other organisms, even though many functionally-linked protein pairs do not have direct physical interactions. In order to test whether the predicted PPIs in STRING correspond to direct physical protein-protein interactions, we should analyze the similarity between these predicted PPIs with several distinct types of protein pairs such as experimental PPIs obtained from two-hybrid assays, protein pairs that belong to the same protein complexes, and protein pairs that catalyze adjacent steps in enzymatic pathways. As these types of additional information are not available for *M. tuberculosis*, we turn to the model organism *S. cerevisiae* where more comprehensive information is available. We extract from STRING an unbiased representative *S. cerevisiae* PPI subset (which we denote “predicted functional associations dataset”) that are predicted using similar methods as the H37Rv STRING PPI dataset. For the three different types of protein pairs, we use the following gold standard: (i) the *S. cerevisiae* two-hybrid PPI dataset from Yu et al [10], (ii) all protein pairs found in the same *S. cerevisiae* protein complexes from Wodak Lab [11], and (iii) protein/gene pairs that catalyze/form successive reaction steps in biological pathways from KEGG [12], WikiPathways [13,14] and BioCyc [15].

This analysis indicates that the predicted *S. cerevisiae* STRING PPIs show higher similarities with protein pairs in the same protein complexes and protein/gene pairs that catalyze/form adjacent reaction steps in biological pathways than with PPIs reported in two-hybrid assays. Therefore, the predicted *S. cerevisiae* STRING PPIs are mostly not direct physical protein-protein interactions. As the H37Rv STRING PPIs are predicted using similar methods, in turn, they are also unlikely to correspond to direct physical interactions. Nonetheless, their relatively good agreement with correlated gene expression profiles, coherent informative GO term annotations, and conservation in other organisms suggest that the H37Rv STRING PPIs (at score ≥ 770) are proteins that are functionally linked.

This work thus provides an important guidance to the researchers who might base their works on the two *M. tuberculosis* H37Rv PPI datasets. The details of our analyses are presented in the sections below.

## Results

This section can be divided into four parts: (i) Discover the low similarity between the two main H37Rv PPI datasets. (ii) Evaluate the quality of the two H37Rv PPI datasets in the same organism. (iii) Assess the quality of the H37Rv B2H PPI dataset across organisms. (iv) Analyse characteristics of the STRING PPIs in *M. tuberculosis* and *S. cerevisiae*.

### Lack of agreement between the two *M. tuberculosis* H37Rv PPI datasets

**Figure 1:**
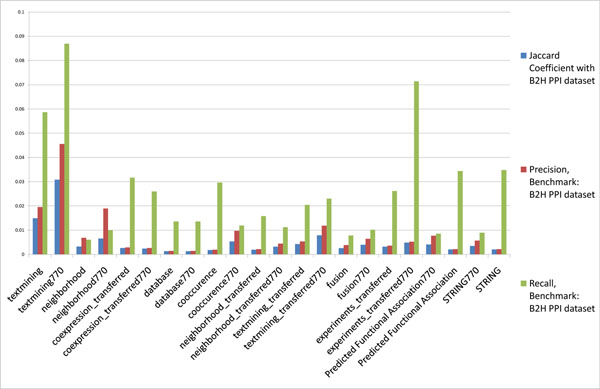
**Agreement between H37Rv PPIs in STRING and the B2H PPI datasets**. The Jaccard coefficient, precision and recall between H37Rv PPI datasets in STRING database predicted by different methods and the H37Rv B2H PPI dataset (benchmark).

**Figure 2:**
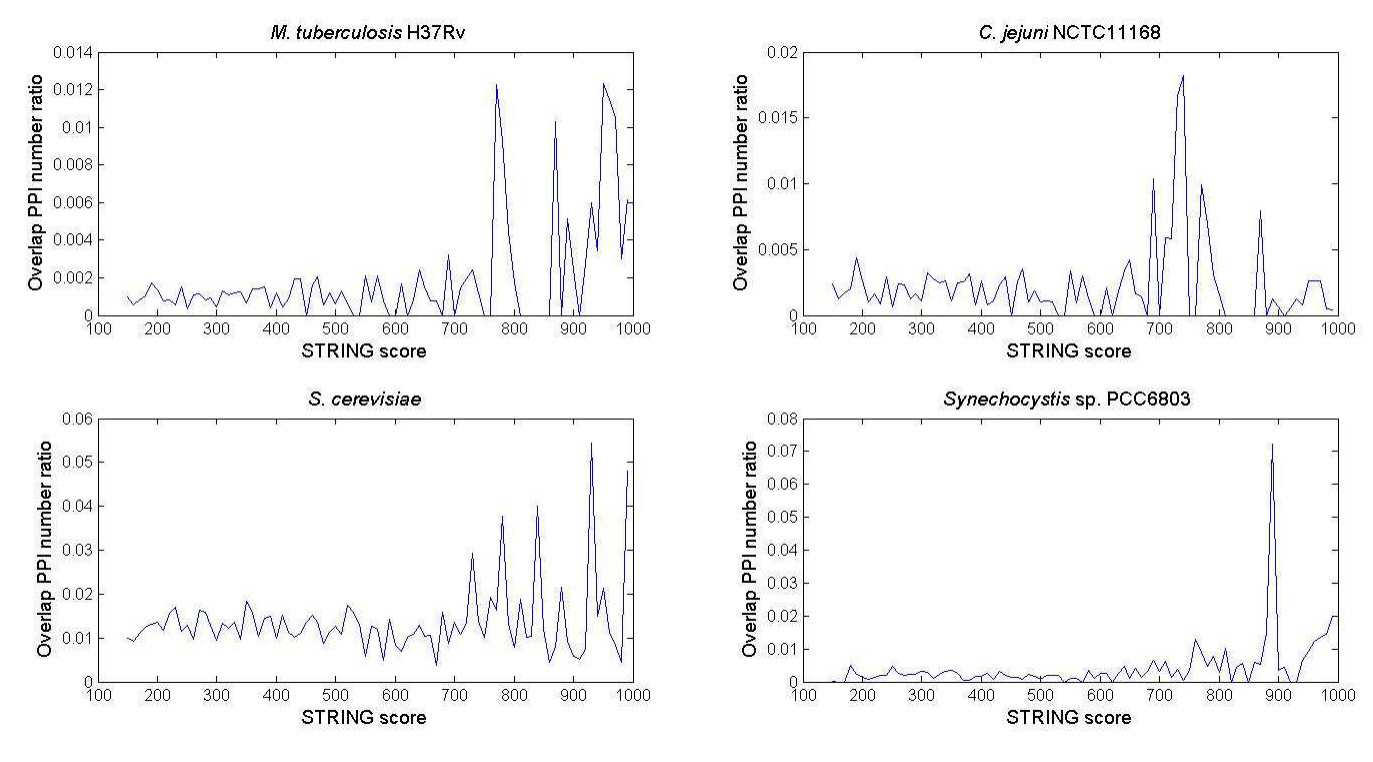
**Overlap PPI number ratios at various STRING score thresholds** The overlap PPI number ratios at various STRING score thresholds between (i) the H37Rv B2H PPI dataset and the H37Rv STRING predicted functional associations dataset, (ii) the *S. cerevisiae* Y2H PPI dataset and the *S. cerevisiae* STRING predicted functional associations dataset, (iii) the *C. jejuni* NCTC11168 Y2H PPI dataset and the *C. jejuni* NCTC11168 STRING predicted functional associations dataset, and (iv) the *Synechocystis* sp. PCC6803 Y2H PPI dataset and *Synechocystis* sp. PCC6803 STRING predicted functional associations dataset.

The H37Rv B2H PPI dataset is used as benchmark and different subsets of H37Rv STRING PPIs are tested against it. We consider all H37Rv STRING PPIs as well as STRING H37Rv PPIs based on specific methods (gene neighbourhood, gene fusion, etc.; details are listed in Table 1) used for predicting them. For each subset, in Figure 1, we show the Jaccard coefficient, precision, and recall of each predicted subset at STRING score ≥ 770. According to Figure 2, STRING score threshold at around 770 generally maximizes the overlap between two-hybrid PPIs and STRING predicted PPIs in *M. tuberculosis* H37Rv. It is clear from Figure 1 that the STRING PPIs predicted by various methods all have extremely low precision, recall, and overlap with the H37Rv B2H PPI dataset. Below are some representative statistics:

**Table 1:** Summary of number of PPIs in each STRING prediction approaches

STRING database prediction method	Number of PPIs	STRING database prediction method	Number of PPIs
Neighbourhood	12,706	Transferred neighborhood	78,376
Co-expression	0	Transferred co-expression	4,393
Experiments	4	Transferred experimental	4,129
Databases	7,030	Transferred databases	629
Text mining	2,715	Transferred text mining	11,074
Gene fusion	2,646	Co-occurrence	159,213

All STRING database PPIs	248,574		

Summary of various subsets of H37Rv PPIs in STRING and their sources.

Overlapping PPIs between the two datasets: 276.

STRING PPIs Precision: 0.00215 Recall: 0.03503.

STRING PPIs (at score ≥ 770) Precision: 0.00574 Recall: 0.00896.

The extremely low agreement between the H37Rv PPIs in the STRING and B2H PPI datasets is rather unexpected. We hypothesize that it may be a result of one or more of the following situations. First, it may be that the H37Rv B2H PPI dataset contains an unusually high level of noise. Second, it may be that the H37Rv STRING PPI dataset and subsets thereof contain an unusually high level of noise. Third, it may be that the predicted PPIs in STRING are not direct physical interactions; rather, they may primarily be other types of functional associations such as protein pairs in the same protein complexes and enzyme pairs catalyzing successive reaction steps.

### Overlap PPI number ratios at various STRING score thresholds

The results above reveal the surprisingly low coverage between the two H37Rv PPI datasets. However, as shown in Figure 2, at STRING score ≥770, there is a higher level of overlap between the two datasets. This increase in overlap between two-hybrid PPI dataset and STRING predicted functional associations dataset at high scores is also observed in *C. jejuni*[8], *Synechocystis *[9] and *S. cerevisiae*[10]. This suggests that STRING PPIs with high score potentially has higher quality than STRING PPIs with a lower score. Nevertheless, the overlap between these two-hybrid PPI datasets and their respective STRING predicted functional association datasets is no more than 8% at any score interval. Thus, even at a high STRING score threshold, there is no clear agreement between two-hybrid PPIs and STRING predicted functional association datasets. Assuming that not all of these two-hybrid PPI datasets are of low quality, this lack of clear agreement strongly suggests that STRING predicted PPIs are unlikely to correspond mainly to direct physical interactions.

### Analysis of PPI dataset using PPI functional intensity matrix

**Figure 3:**
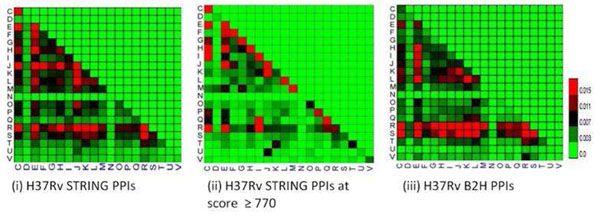
**PPIs functional intensity matrix analysis of the PPI datasets.** Functional intensity matrices of (i) H37Rv STRING PPI dataset, (ii) H37Rv STRING PPI dataset at score ≥ 770, and (iii) H37Rv B2H PPI dataset. The “C”, “D”, “E”, etc. in the axes of these matrices are COG functional categories.

We next use PPI functional intensity matrix to visualize the functional relationship between the two interacting proteins in PPIs. Figure 3 shows that the H37Rv STRING PPI dataset (with score ≥ 770) has strong intensity at the diagonal of its PPI functional intensity matrix. This means a substantial portion of PPIs in this dataset have both partners in the same function category. In contrast, both the H37Rv B2H PPI dataset and the H37Rv STRING PPI dataset (without thresholding at score ≥ 770) exhibit weak intensity at the diagonal of its matrix. Moreover, both the H37Rv B2H PPI dataset and the H37Rv STRING PPI dataset (without thresholding at score ≥ 770) have substantial amounts of PPIs involving proteins that are functionally unclear or uninformative.

### Assessment of PPI datasets using informative GO terms

The functional intensity matrix visualisation in Figure 3 only provides broad perspectives on the functional distribution of PPI datasets. It is not sufficient for gauging the quality of the datasets. Two interacting proteins are more likely to be localized in the same cellular component and/or having a common function role than not [16]. So we calculate the percentage of PPIs in a PPI dataset having coherent informative GO terms—i.e., the rate of interacting protein pairs with common function roles (measured based on informative GO terms in MF and BP categories) and cellular localization (measured based on informative GO terms in the CC category) in the PPI dataset—to evaluate the quality of the PPI dataset.

**Figure 4:**
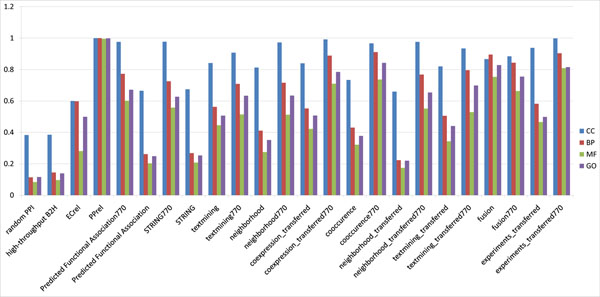
**Percentage of PPIs in various *M. tuberculosis* PPI datasets that have coherent informative GO term annotations.** Percentage of PPIs in various *M. tuberculosis* PPI datasets that have coherent informative GO term annotations.

The results of *M. tuberculosis* H37Rv GO term annotation and informative GO term identification are summarized in Table 2. The percentage of PPIs having coherent informative GO terms is computed for each of the datasets in Figure 4. The datasets include subsets of STRING derived from specific source channels in STRING. Note that some source channels may introduce GO-related information into STRING. In particular, the “database” and “database transfer” channels may collect PPIs derived from Protein Complexes in the Gene Ontology (GO) database. Thus, to avoid circularity in our results here and elsewhere, we mainly use statistics from an unbiased subset “predicted functional associations dataset” of STRING obtained by excluding the PPIs from source channels that may introduce confounding factors. The “predicted functional associations dataset” consists of STRING PPIs that are generated only from the following prediction approaches: gene neighborhood, transferred neighborhood, gene fusion, and co-occurrence, transferred co-expression, text mining, and transferred text mining. Among the three categories of GO terms, the datasets generally show a high percentage of coherence with respect to informative CC GO terms. However, this observation should be dismissed because there are only three distinct informative CC GO terms, which is an order of magnitude less than informative MF and BP GO terms; see Table 2. The random PPI dataset has the lowest percentage of PPIs with coherent informative GO terms in all the tested PPI datasets, which makes sense. But the H37Rv B2H PPI dataset has the second lowest percentage of PPIs with coherent informative GO terms and is very close to the random PPI dataset. This indicates that the *M. tuberculosis* H37Rv B2H PPI dataset has the lowest quality among all the PPI datasets been evaluated. The H37Rv predicted functional association dataset (without thresholding at score ≥ 770) also has a low percentage of PPIs with coherent informative GO terms and is thus of low quality. However, most PPI datasets from STRING (with score ≥ 770) show a much higher percentage of PPIs having coherent informative GO terms than the H37Rv B2H PPI dataset, suggesting that a higher percentage of PPIs in these datasets may have better reliability than those of the B2H PPI dataset and of the STRING PPI dataset as a whole.

**Table 2:** *M. tuberculosis* H37Rv GO term annotation and informative GO term identification

	No. of informative GO term	No. of proteins been annotated with GO term	No. of proteins been annotated with informative GO term (N = 30)
CC	3	563	233
BP	32	1822	1200
MF	32	2034	1198
GO	67	2225	1659

This table summarizes the result of *M. tuberculosis* H37Rv GO term annotation and informative GO term identification.

### Analysis of PPI datasets using gene expression profile correlation

**Figure 5:**
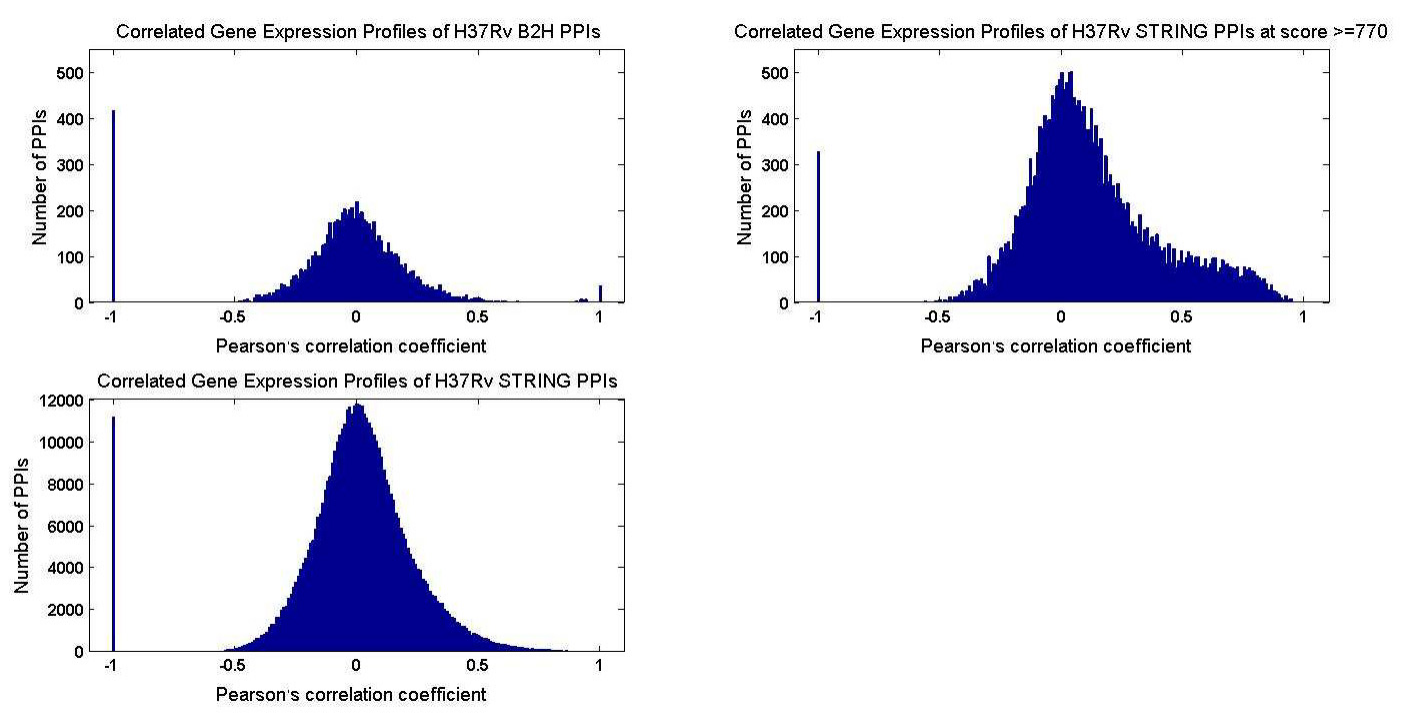
**PPI datasets assessment by gene expression profile correlation.** The distribution of Pearson’s correlation coefficient of the expression profiles of underlying genes of different PPI datasets are given in this figure (x axis is the Pearson’s correlation coefficient, y axis is the number of PPIs). The bar at -1 in the charts here corresponds to PPIs where we do not have the expression profiles of their underlying genes.

Two proteins that interact are more likely to be correlated in the expression of their underlying genes than not [17]. In fact, co-expression is one of the prediction methods in STRING [3]. However, Table 1 shows no STRING PPI predicted from co-expression in *M. tuberculosis* H37Rv. Given that *M. tuberculosis* H37Rv gene expression data is readily available in public repositories (e.g., NCBI Gene Expression Omnibus [18]), this lack of H37Rv PPIs predicted using this information is an unexpected limitation of STRING. At the same time, this absence makes using correlation of gene expression profiles for assessing the quality of the H37Rv B2H and STRING PPI datasets unbiased. The results in Figure 5 clearly show that the H37Rv STRING PPI dataset (at score ≥ 770) has a much larger proportion of PPIs that exhibit correlation in the expression profiles of their underlying genes than the H37Rv B2H PPI dataset and the whole H37Rv STRING PPI dataset. In fact, a mere 223 PPIs in the H37Rv B2H PPI dataset have significant correlated gene expression profiles (Pearson’s correlation coefficient >0.4). These 223 PPIs are likely to be more reliable than most of the other PPIs in the H37Rv B2H dataset.

### PPI datasets assessment using predicted interologs from STRING experimental PPIs

As PPIs are often conserved by related organisms, homology transfer is an often-used technique to predict PPIs—the so-called interologs. On the one hand, a higher level of agreement that a PPI dataset has with interologs gives us confidence that the PPIs are more reliable—i.e., fewer false positives—as they are conserved in other organisms. On the other hand, a lower level of agreement signals that PPIs conserved in related organisms are potentially missing—i.e., more false negatives—in the PPI dataset.

We use different homology transfer methods to build a set of predicted interologs in the target organism *M. tuberculosis* H37Rv from other (non-*M. tuberculosis*) organisms that have experimental PPIs in STRING. We then compare this set of interologs to both the H37Rv STRING predicted functional associations dataset (with score ≥ 770) and H37Rv B2H PPI dataset. We use this subset of STRING PPIs (predicted functional associations dataset) to avoid possible confounding effects because the full set of H37Rv STRING PPIs contains some PPIs that actually originate from interologs predicted from experimental PPIs.

In order to find better source PPIs for predicting interologs, a more reliable subset of the experimental PPIs in STRING has been chosen in this experiment. This subset consists of the experimental PPIs in STRING that also have STRING prediction supports.

Table 3 shows the results of interologs predicted from STRING experimental PPIs, while Table 4 shows the results of interologs predicted from the reliable subset of STRING experimental PPIs. Under the same method and benchmark, Jaccard coefficient, precision and recall in Table 4 are much higher than that in Table 3. This demonstrates STRING experimental PPIs with prediction supports is a better source than the whole STRING experimental PPIs for predicting interologs. Because experimental PPIs also have significant noise in most organisms, choosing the subset with both experiments and STRING prediction supports would have better reliability. When switching benchmark from the H37Rv B2H PPI dataset to the H37Rv STRING predicted functional associations dataset (with score ≥770), the resulting Jaccard coefficient, precision, recall, and overlapping PPIs number, increase significantly (about two orders of magnitude). This clearly demonstrates that the predicted *M. tuberculosis* H37Rv interologs from other (non-*M. tuberculosis*) organisms’ experimental PPIs in STRING are in much lower agreement with the H37Rv B2H PPI dataset than with the H37Rv STRING predicted functional associations dataset (with score ≥ 770). In particular, depending on the method used for constructing interologs, just 4-5 interologs are found in the H37Rv B2H PPI dataset, while 524-830 interologs are found in the H37Rv STRING predicted functional associations dataset (with score ≥ 770); see Table 4. This suggests that the H37Rv B2H PPI dataset may have a high number of false negatives.

**Table 3:** Results of predicted interologs from STRING experimental PPIs

Source PPIs for homology transfer	STRING database experimental PPIs		
Identify homology and transfer	PIDE >30	HVAL >20	Orthologues
	Coverage >0.2	E-VALUE <1*e-6	Identified by Inparanoid (default parameters)
	E-VALUE< 1*e-10		

Benchmark	*M.tuberculosis* H37Rv high-throughput B2H PPIs dataset		

Jaccard coefficient	0.00354	0.00187	0.00289
Precision	0.00375	0.00196	0.00339
Recall	0.0588	0.0384	0.0190
Overlapping PPIs	5	4	5

Benchmark	*M.tuberculosis *H37Rv predicted functional associations dataset from STRING database(with PPIs score above 770)		

Jaccard coefficient	0.285	0.179	0.129
Precision	0.355	0.236	0.239
Recall	0.625	0.422	0.219
Overlapping PPIs	656	830	525

Predicted interologs from STRING experimental PPIs and a summary of Jaccard coefficient, precision and recall between the interologs datasets and two benchmarks.

**Table 4:** Results of predicted interologs from STRING experimental PPIs (with other STRING database prediction support)

Source PPIs for homology transfer	STRING database experimental PPIs (with other prediction support)		
Identify homology and transfer	PIDE >30	HVAL >20	Orthologues
	Coverage >0.2	E-value <1*e-6	Identified by inparanoid (default parameters)
	E-value< 1*e-10		

Benchmark	*M.tuberculosis *H37Rv high-throughput B2H PPIs dataset		

Jaccard coefficient	0.00485	0.00225	0.00359
Precision	0.00507	0.00267	0.00401
Recall	0.100	0.0563	0.0331
Overlapping PPIs	5	4	4

Benchmark	*M.tuberculosis *H37Rv predicted functional associations datasetfrom STRING database (with PPIs score above 770)		

Jaccard coefficient	0.365	0.233	0.186
Precision	0.467	0.314	0.365
Recall	0.625	0.473	0.275
Overlapping PPIs	651	828	524

Predicted interologs from STRING experimental PPIs (with other STRING database prediction support) and a summary of Jaccard coefficient, precision and recall between the interologs datasets and two benchmarks.

### PPI datasets assessment using predicted interologs from IntAct prokaryotic physical interactions

The results discussed above are already persuasive but not precise enough to make sufficient judgment. In particular, the experimental PPIs in STRING are a mixture of both experimental physical interactions and experimental functional associations (e.g., genetic interactions). This may introduce some bias towards the larger agreement observed with the STRING predicted functional associations dataset. Furthermore, some source experimental PPIs in the dataset are not from organisms close to *M. tuberculosis*. To control for these potential confounding factors, we conduct another set of experiments using interologs predicted from the following two sources that—in theory—are closer to the characteristics of H37Rv B2H PPIs. The first source is the latest pull-down PPI dataset in *S. aureus* MRSA252 [19]. This dataset has just been released and is not yet included in the current STRING database (version 8.3). The second source comprises physical interactions of eight bacteria (mostly pathogens) in the IntAct database [20]. In particular, we have chosen *Rickettsia sibirica* 246, *Escherichia coli* K12, *Campylobacter jejuni*, *Treponema pallidum*, *Synechocystis* sp. PCC 6803, *Mycoplasma pneumonia*, *Myxococcus xanthus* DK 1622, and *Streptococcus pneumoniae*. We group the physical interactions of these eight bacteria from IntAct [20] into the “selected IntAct prokaryotic physical interactions dataset”.

We transfer interologs from the “selected IntAct prokaryotic physical interactions dataset” to *M. tuberculosis* H37Rv (named “H37Rv physical interologs dataset”) and to *S. aureus* MRSA252 (named “MRSA252 physical interologs dataset”). We also transfer interologs from the *S. aureus* MRSA252 high-throughput pull-down PPI dataset to *M. tuberculosis* H37Rv (named “H37Rv pull-down interologs dataset”). All the homolog identifications of this experiment use the condition PIDE >30, Coverage >0.2, E-VALUE< 1*e-10.

The comparative analyses results are given in Table 5. The results clearly demonstrate that the H37Rv physical interologs dataset is much more similar to the H37Rv pull-down interologs dataset than to the H37Rv B2H PPI dataset. Moreover, the similarity between *S. aureus* MRSA252 physical interologs dataset and pull-down PPI dataset is also much higher than that between H37Rv physical interologs dataset and H37Rv B2H PPI dataset. These results further strengthen the conclusion that the H37Rv B2H PPI dataset has poor quality.

**Table 5:** Comparison of physical interactions and predicted physical interologs datasets

Organism	Benchmark dataset	Testing PPI dataset	Jaccard coefficient	Precision	Recall	Overlapping PPIs No.
*M. tuberculosis* H37Rv	H37Rv pull-down interologs	H37Rv physical interologs	0.021	0.205	0.023	40
*M. tuberculosis* H37Rv	H37Rv B2H PPI	H37Rv physical interologs	0.005	0.005	0.050	2
*S. aureus* MRSA252	MRSA252 Pull Down PPI dataset	MRSA252 physical interologs	0.019	0.126	0.022	23

Summary of the comparison results between physical interactions and predicted interologs datasets. “H37Rv physical interologs dataset” consists of interologs transferred from “selected IntAct prokaryotic physical interactions dataset” to *M. tuberculosis* H37Rv; “MRSA252 physical interologs dataset” consists of interologs transferred from “selected IntAct prokaryotic physical interactions dataset” to *S. aureus* MRSA252; “H37Rv pull-down interologs dataset” consists of interologs transferred from “*S. aureus* MRSA252 high-throughput pull-down PPI dataset” to *M. tuberculosis* H37Rv.

### Quality evaluation of two-hybrid PPI datasets from several organisms

**Figure 6:**
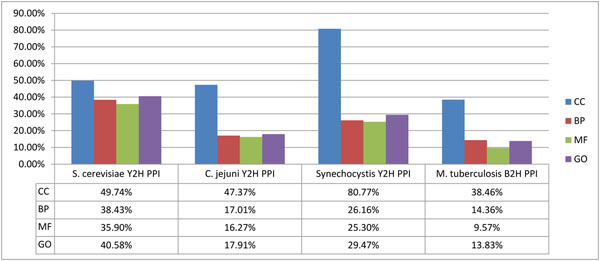
**Percentage of PPIs in representative two-hybrid PPI datasets having coherent informative GO terms**. The histogram is a summary of the percentage of PPIs having coherent informative GO terms in each of the representative two-hybrid PPI datasets in four organisms.

In the discussions above, we have concluded that the quality of the *M. tuberculosis* H37Rv B2H PPI dataset is low in quality. However, it is generally believed that two-hybrid PPI datasets do not have high reliability because of their high false positives and false negative rates. It is thus of interest to determine whether this H37Rv B2H PPI dataset is even worse than other two-hybrid PPI datasets in terms of quality. So we compare the *M. tuberculosis* H37Rv B2H PPI dataset to several representative two-hybrid PPI datasets in other organisms*—*viz., *C. jejuni*, *Synechocystis* and *S. cerevisiae*[8-10]*—*by calculating their percentage of PPIs having coherent informative GO terms. The results are shown in Figure 6. As these organisms have a large difference in the number of informative GO terms and in the distribution of GO term annotations to their proteins, we also calculate the ratio of the percentage of coherently annotated PPIs in the dataset of an organism to that of appropriately generated random PPI datasets. The results are shown in Table 6. In particular, the ratios for *M. tuberculosis*, *C. jejuni*, *Synechocystis* and *S. cerevisiae* are respectively 1.20, 1.28, 3.03, and 5.73. These results sufficiently demonstrate that, compared to other representative two-hybrid PPI datasets, the *M. tuberculosis* H37Rv B2H PPI dataset has distinctly lower percentage of coherently annotated PPIs.

**Figure 7:**
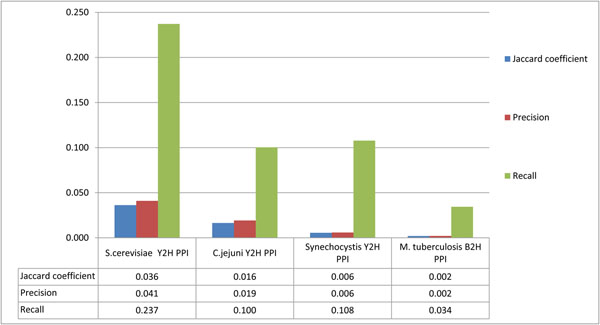
**Similarity of representative two-hybrid PPI datasets to corresponding STRING predicted functional associations datasets.** The histogram is a summary of calculated Jaccard coefficient, precision and recall between a representative two-hybrid PPI dataset (as benchmark) and its corresponding STRING predicted functional associations dataset (as testing dataset).

We have also computed for each two-hybrid PPI dataset its similarity to the corresponding predicted functional associations dataset from STRING; see Figure 7. These results indicate the H37Rv B2H PPI dataset also has the lowest similarity to the corresponding STRING predicted functional association dataset.

**Table 6:** Percentage of PPIs in representative two-hybrid PPI datasets having coherent informative GO terms

	CC	BP	MF	GO
*S. cerevisiae* Y2H PPI	49.74%	38.43%	35.90%	40.58%
*S. cerevisiae* random PPI	5.03%	3.70%	7.72%	7.08%
Info GO ratio of *S. cerevisiae* (Y2H PPI/ random)	9.88	10.38	4.65	5.73
Info GO term No.	57	173	78	365
*C. jejuni* Y2H PPI	47.37%	17.01%	16.27%	17.91%
*C. jejuni* random PPI	35.61%	11.04%	12.91%	13.97%
Info GO ratio of *C. jejuni* (Y2H PPI / random)	1.33	1.54	1.26	1.28
Info GO term No.	3	26	22	51
*Synechocystis* Y2H PPI	80.77%	26.16%	25.30%	29.47%
*Synechocystis* random PPI	44.94%	6.89%	8.59%	9.73%
Info GO ratio of *Synechocystis* (Y2H PPI / random)	1.80	3.79	2.95	3.03
Info GO term No.	3	30	30	63
*M. tuberculosis* B2H PPI	38.46%	14.36%	9.57%	13.83%
*M. tuberculosis* random PPI	38.30%	11.35%	8.35%	11.51%
Info GO ratio of *M. tuberculosis* (B2H PPI / random)	1.00	1.26	1.15	1.20
Info GO term No.	3	32	32	67

The table is a summary of the percentage of PPIs having coherent informative GO terms, number of informative GO terms and “Info GO ratio” in each of the representative two-hybrid PPI datasets in four organisms.

Info GO ratio = percentage of PPIs in two-hybrid PPI dataset having coherent informative GO terms / percentage of PPIs in random PPI dataset having coherent informative GO terms.

### Analysis of the characteristics of *M. tuberculosis* H37Rv PPIs using integrated pathway gene pair relationships

From the results presented earlier, it seems that many H37Rv STRING PPIs may not be direct physical interactions. In order to understand what these PPIs may better correspond to, we collect pair-wise protein/gene relationships (mainly the ECrel and PPrel datasets, see Table 7) from several major pathway databases, and compare them with the various PPI datasets considered earlier in this paper. The ECrel dataset comprises enzyme pairs that catalyze successive reaction steps in enzymatic pathways. The PPrel dataset comprises more direct protein-protein interactions but it also contains protein pairs in the same complexes. Thus, a PPI dataset containing more indirect protein relationships should show high similarity to the ECrel dataset.

**Table 7:** Four types of gene relationships in integrative pathway gene pair relationships

Unified genes relationships	Explanation
ECrel	Enzyme-enzyme relation, indicating two enzymes catalyzing successive reaction steps
PPrel	Protein-protein interaction, such as binding and modification, or proteins belong to same complex
GErel	Gene expression interaction, indicating relation of transcription factor and target gene product
PCrel	Protein-compound interaction

The detail explanations of the types of relationships in integrated pathway gene pair relationships are given below.

However, this task is hampered by the sparse information stored in all the current main pathway databases, like KEGG [12], WikiPathways [13,14] and BioCyc [15]. Therefore an integration of pathway information from the three main databases is needed to maximize the effectiveness of pathway information for this comparative analysis of PPI datasets.

The number of pathways obtained from the three major pathway databases [12-15], and the number of pathways after integration are listed in Table 8. All together, the *M. tuberculosis* H37Rv integrated pathway gene pair relationships contain 230 pathways, comprising 3,573 gene pairs involving 668 proteins.

**Table 8:** Summary of number of pathways before and after integration

Pathways source name	No. of pathways *before* integration	No. of unique pathways *after* integration
WikiPathways	5 pathways	1 pathways
BioCyc (MTBRVcyc)	191 pathways	129 pathways
KEGG	80 pathways	66 pathways
Integrated Pathways		34 pathways

The table below shows the number of pathways from major pathway databases before and after integration.

**Figure 8:**
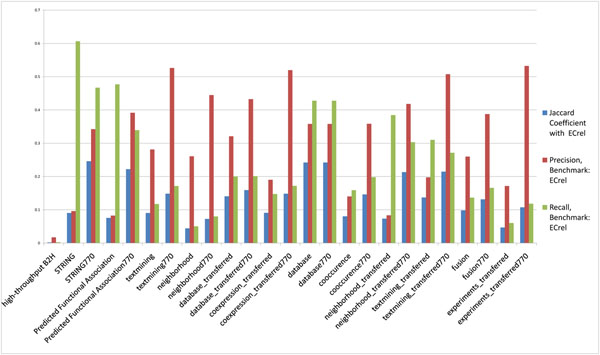
**Comparative analysis of PPI datasets using integrated pathway gene pair relationships (ECrel). ***M. tuberculosis* H37Rv PPI datasets similarity to integrated pathway gene pair relationships (ECrel dataset as benchmark).

**Figure 9:**
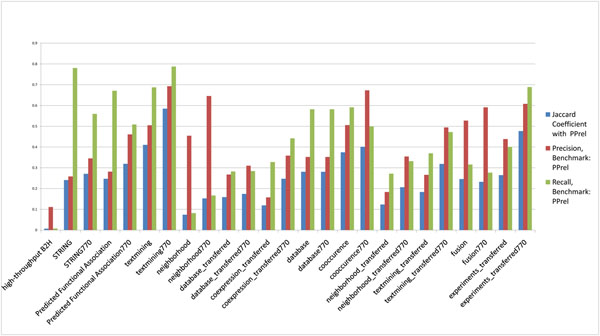
**Comparative analysis of PPI datasets using integrated pathway gene pair relationships (PPrel). ***M. tuberculosis* H37Rv PPI datasets similarity to integrated pathway gene pair relationships (PPrel dataset as benchmark).

In the *M. tuberculosis* H37Rv integrated pathway gene pair relationships, most of the gene pair relationships are ECrel (2,527 pairs) and PPrel (916 pairs), other type of relationships are very few. We calculate the Jaccard coefficient, precision, and recall of each of the *M. tuberculosis* H37Rv PPI datasets discussed earlier using ECrel (Figure 8) and PPrel (Figure 9) from *M. tuberculosis* H37Rv integrated pathway gene pair relationships as the benchmarks.

Results from above experiments show that the *M. tuberculosis* H37Rv B2H PPI dataset shows very low similarity with ECrel dataset, while most of STRING PPI datasets show good similarity. This provides another explanation for the low similarity between the H37Rv B2H and STRING PPI datasets. Namely, the former dataset contains direct physical interactions, as it is to be expected of B2H assays; while the latter STRING datasets also include substantial amounts of PPIs that are indirect protein relationships*.*

### STRING PPI dataset analysis in *S. cerevisiae*

The comparative analysis of the various H37Rv PPI datasets using integrated pathway gene pair relationships reveals that the H37Rv STRING PPI dataset may contain a lot of indirect protein relationships. The STRING database has proclaimed itself as a database consisting of “known and predicted protein-protein interactions” [21]. In practise, both physical interactions and functional associations, and both predicted and experimental “PPIs” are included in this database. Therefore, it is important to clearly demonstrate which kind of PPIs are contained in STRING. We return to the most comprehensively investigated model organism—*S. cerevisiae —*to more precisely analyze the characteristics of PPIs in STRING. As a unified database, the PPIs prediction approaches in the STRING database are consistently used on all the 630 organisms included in it. Thus the phenomena discovered in *M. tuberculosis* H37Rv should also exist in other organisms like *S. cerevisiae*, and vice versa. Moreover, we have much more information in *S. cerevisiae* that can be used for conducting a much more precise analysis. If the situation observed earlier that the *M. tuberculosis* H37Rv STRING PPI dataset contains a lot of indirect PPIs is also observe in *S. cerevisiae*, then it will be a sound confirmation of our earlier conclusion.

We similarly obtain the integrated pathway gene pair relationships (mainly ECrel and PPrel) for *S. cerevisiae* and also separate datasets prepared only from KEGG [12] for more precise reference. We further collect all protein pairs (named the “*S. cerevisiae* Complex PPI dataset”) that appear in the same protein complexes using the protein complexes dataset from Wodak Lab [11]. It is obvious that the “*S. cerevisiae* Complex PPI dataset” may contain a lot of indirect PPIs, like relationships between two non-directly-binding proteins in protein complexes. A representative *S. cerevisiae* two-hybrid PPI dataset [10] is also included in this comparative analysis.

**Figure 10:**
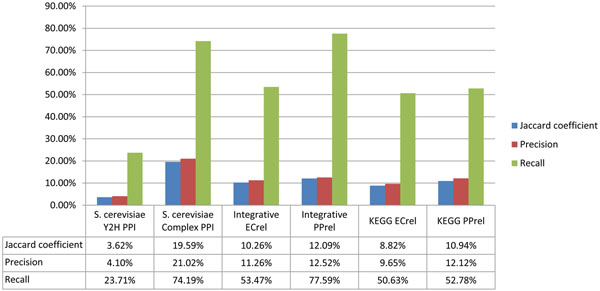
**Comparative analysis of different *S. cerevisiae* protein relationships datasets with *S. cerevisiae* STRING functional associations dataset.** Comparison of the similarity between different protein relationships datasets with *S. cerevisiae* predicted functional associations from STRING database.

To avoid a biased comparison, as the full STRING PPI dataset may include many PPIs from the datasets above, we use the *S. cerevisiae* predicted functional associations dataset from STRING database as the testing dataset in this analysis. The overlapping number of PPIs, Jaccard coefficient, precision and recall are calculated, and the results are given in Figure 10. From the results, the *S. cerevisiae* two-hybrid PPI dataset has the lowest similarity to the *S. cerevisiae* STRING predicted functional associations dataset, whereas the complex PPI dataset and ECrel datasets (both from KEGG and from integrated pathway gene pair relationships) reveal good similarity to the *S. cerevisiae* STRING predicted functional associations dataset. This result is in accordance with the results on *M. tuberculosis* H37Rv, clearly demonstrating that the STRING database PPIs include a substantial amount of PPIs that are indirect protein relationships, including protein pairs in the same protein complexes and protein pairs catalyzing successive enzymatic reaction steps.

## Discussion

### Reliable *M. tuberculosis* H37Rv B2H PPI datasets

We have shown that the *M. tuberculosis* H37Rv B2H PPI dataset has low quality. In the process, we find four subsets of the B2H PPI dataset that may be more reliable than the rest of this dataset. The first subset consists of PPIs where both interaction partners have coherent informative GO terms—viz., B2H PPIs sharing functional homogeneity or localization coherence. This subset contains 115 PPIs and is named “B2HsameGO dataset”. The second contains overlapping PPIs between the H37Rv B2H and STRING PPI datasets, which can be considered as B2H PPIs supported by STRING prediction approaches. This subset consists of 276 PPIs and is named “B2HSTRING dataset”. The third subset contains those H37Rv B2H PPIs that have been verified by different experiments [7]. This subset has 147 PPIs and is named “B2HdiffExp dataset”. The fourth subset contains PPIs where both interaction partners have significant correlated gene expression profile (Pearson’s correlation coefficient > 0.4). This subset has 223 PPIs and is named “B2Hco-express dataset”. The overlap between any pair of these four subsets is small because the PPIs in these four subsets involve very different proteins; see Table 9. When restricted to PPIs with both interaction partners contained in two subsets being compared, the agreements are considerably high, see also Table 9. See Additional file 1 for these reliable *M. tuberculosis* H37Rv B2H PPI datasets.

**Table 9:** Comparative analysis of four reliable H37Rv B2H PPI subsets

Benchmark (b)	Dataset (b) PPIs No.	Testing (t)	Dataset (t) PPIs No.	Overlap PPI No.	Jaccard coefficient	Precision	Recall	Benchmark PPI No.	Testing PPI No.
B2Hco-express	223	B2HsameGO	115	17	0.85	0.89	0.944	18	19
B2Hco-express	223	B2HSTRING	276	27	0.44	0.77	0.5	54	35
B2Hco-express	223	B2HdiffExp	147	4	0.16	0.27	0.29	14	15
B2HsameGO	115	B2HdiffExp	147	0	0	Null	0	1	0
B2HsameGO	115	B2HSTRING	276	11	0.48	0.73	0.58	19	15
B2HSTRING	276	B2HdiffExp	147	6	0.16	0.19	0.55	11	31

The table is a summary of brief comparative analysis of four reliable H37Rv B2H PPI subsets.

There are several inherent limitations of PPI data generated by two-hybrid approaches (both B2H and Y2H), including PPIs that are detected between over-expressed proteins, between fusion proteins, and in a different host (Yeast or *E. coli*). Given the data available in this study, we are not able to clearly identify which erroneous B2H PPIs are caused by which inherent limitations of the two-hybrid system. We leave this interesting and difficult challenge to a future project.

### Differences between functional associations and physical interactions

Physical interactions correspond to direct protein relationships like two proteins binding to each other. Functional associations can be both direct and indirect protein relationships; for example, two enzymes catalyzing successive reaction steps can be regarded as functional associations. This partially explains the differences between the H37Rv B2H and STRING PPI datasets, as we have demonstrated earlier.

Based on the approach used in generating the PPIs, each of the two major categories can be further divided into two parts, “**experimental** physical interactions” (e.g., PPIs from Y2H or co-purification); “**predicted** physical interactions” (e.g., interologs predicted from co-purification PPIs); “**experimental** functional associations” (e.g., PPIs from synthetic lethality or dosage growth defect); “**predicted** functional associations” (e.g., PPIs from neighbourhood or co-occurrence). Differences among PPI datasets from the four categories are inevitable, and they all have some portion of real PPIs and some noise. However, a high noise level often overwhelms the agreement between the datasets from these four categories. Real PPIs are both functional associations and physical interactions (intersect dataset), because if two proteins truly interact with each other in normal environments, the two proteins must have functional relationships. The four subsets of reliable H37Rv B2H PPIs identified by us only contain a small number of PPIs and are not enough to illuminate the whole direct physical interactome in *M. tuberculosis* H37Rv, See Additional file 1 for the four subsets of reliable H37Rv B2H PPIs. Although we have identified a reliable subset of STRING PPI dataset (with score ≥ 770) it may contain a substantial amount of PPIs that are indirect functional associations. Some approaches [22-24] used for protein complex prediction from PPI data can potentially be used to identify physical interactions from STRING functional associations. We leave this interesting problem to a future project.

### Comments on the STRING database

The H37Rv STRING PPI dataset (with score ≥ 770) has its merits, especially with respect to functional associations as described earlier. However, some obvious limitations of STRING still need to be overcome in order to maximize its benefits to the community.

Firstly, STRING version 8.3 does not make the source of individual PPIs completely transparent to the public. Neither the web interface nor the downloadable “protein.link.detail.v8.3.txt” file provides a clear indication of a PPI’s exact source, especially for users who lack a license for accessing the “protein.link.full.v8.3.txt” file. For example, they can only know that a PPI comes from “co-expression” but it can actually either come from “transferred co-expression” in other organisms through homology transfer or from “co-expression” of this organism. When looking at the “protein.link.detail.v8.3.txt” file, the H37Rv STRING PPI dataset contains 4,133 PPIs from experiments, whereas only 4 of which actually come from experiments in H37Rv and the rest are from “transferred experiments”. When users have the license to access all the information in this database, they still do not have a clear sense of what type of PPIs are included in STRING. It claimed to consist of “known and predicted protein-protein interactions” on its website [21], though in its publications it emphasizes on “functional associations” [3]. Actually, both “physical interactions” and “functional associations”, and both “predicted” and “experimental” PPIs, are included in this database. It is helpful to collect and combine all the PPIs in the STRING database; but users should be informed of each PPI’s source (predicting or reporting approaches) and characteristics (physical or functional).

Second, each version of the STRING database release time is quite long, making the STRING PPI datasets incomplete and often obsolete for many organisms. Although some PPI datasets have been published months ago, like the *S. aureus* MRSA252 pull-down PPI dataset [19] and the *M. tuberculosis* H37Rv B2H PPI dataset [7], neither of them has been included in the STRING database current version 8.3. The difficulty of keeping the PPI data of every organism updated in this large database (containing some 630 organisms) in a timely efficient manner is understandable. We suggest the STRING database team to divide the organisms included in the database into several tiers—for tier-1 organisms, frequent updates should be maintained; for tier-2 organisms, maybe less frequent updates are needed; and so on. This differential update process within the same large database should overcome this limitation and maximize the benefits of the STRING database.

### Compare the quality of high-throughput PPI datasets indirectly

Ideally, we should have used confirmed PPI data as the benchmark in evaluating the quality of the H37Rv B2H and STRING datasets. Unfortunately, such confirmed PPI data is not available in a sufficiently large scale for *M. tuberculosis* H37Rv. Hence, we have resorted to the rather indirect evidence presented in this paper.

Due to inherent limitations of two-hybrid approaches, both B2H- and Y2H-generated PPI datasets usually have low quality. It is more appropriate to compare the quality of the *M. tuberculosis* H37Rv B2H PPI dataset with that of other B2H PPI datasets; but such large-scale reference B2H PPI datasets are currently not available in public databases. However, by comparing the quality of the B2H PPI dataset with the quality of other representative two-hybrid PPI datasets, we can still conclude that, besides inherent limitations of two-hybrid approaches, the H37Rv B2H PPI dataset has lower quality than other two-hybrid PPI datasets. This suggests that the noise introduced by individual experiments in this H37Rv B2H PPI dataset may be higher than other datasets compared.

Hopefully, in the near future, more high quality B2H PPI data will be reported and more *M. tuberculosis* H37Rv gold standard PPI data will be confirmed. We will then be in more suitable position to address this concern in a more direct and effective way.

## Conclusions

In this work, we have observed the strikingly low agreement between *M. tuberculosis* H37Rv B2H and STRING PPI datasets. We have demonstrated the two main causes of this low level of agreement. The first reason is the low quality of the B2H PPI dataset, which seems to contain a significant amount of false positives as well as false negatives. The same is true of the H37Rv STRING PPI dataset as a whole, though a subset comprising PPIs with score ≥ 700 seems more reliable. The second reason is that the STRING PPI dataset contains a substantial amount of predicted PPIs that are not direct interactions, which seem more likely to correspond to protein pairs that are in the same protein complexes or protein pairs that are catalyzing adjacent reaction steps in enzymatic pathways.

Because of the low quality of the H37Rv B2H PPI dataset, it should not be used as a gold standard to evaluate the quality of other *M. tuberculosis* PPI datasets, predicted or otherwise. Researchers who need to use this dataset should do so with great caution. Yet, as the only available large-scale physical interaction dataset of *M. tuberculosis* H37Rv at the moment, even though it suffers from high noise and low quality, the direct protein physical interaction information in this dataset should not be ignored. We have identified four subsets of this B2H PPI dataset that are more reliable, which can be combined into a single dataset, which can serve as a suitable reference H37Rv physical interaction dataset for many applications.

STRING score is useful for indicating which STRING PPIs have higher quality. We suggest a STRING score threshold set around 770. Nevertheless, the H37Rv STRING PPI dataset (with score ≥ 770) may contain a lot of indirect protein relationships attributable to protein pairs in the same protein complexes or protein pairs forming successive reaction steps in the same biological pathways. Therefore, this dataset can be a good source as a functional associations reference, but it may not be the ideal choice for the purpose of studying physical interactions in *M. tuberculosis* H37Rv.

## Methods

### Preparing STRING PPI datasets for analyses

STRING database uses a combination of prediction approaches and an integration of other information (neighborhood, transferred neighborhood, gene fusion, text mining, databases, homology transfer, co-occurrence, experiments and so on). The details of PPIs generated by each of the approaches are listed in Table 1.

STRING PPIs come from a mix of experimental data; PPIs copied from public databases (e.g. KEGG and BioGRID) and predicted PPIs. So we derive from STRING a subset of predicted PPIs and name this unbiased STRING subset “predicted functional associations dataset”. This dataset is derived only from the following prediction approaches: neighborhood, transferred neighborhood, gene fusion, co-occurrence, transferred co-expression, text mining, and transferred text mining.

### The agreement between a benchmark PPI dataset and a testing PPI dataset

We use Jaccard coefficient, recall, and precision to measure the agreement between a benchmark PPI dataset and a testing PPI dataset. Jaccard coefficient is defined as the size of the intersection of the two datasets divided by the size of the union of the two datasets. Recall is the proportion of benchmark PPIs that are in the testing dataset. Precision is the proportion of testing PPIs that are in the benchmark dataset. Thus,

Jaccard coefficient = TP / (TP+FP +FN);

Precision = TP / (TP+FP);

Recall = TP / (TP + FN).

Here, TP (true positives) represents the number of PPIs in the testing dataset that overlap with the benchmark dataset; FN (false negatives) represents the number of PPIs in the benchmark dataset that are not in the testing dataset; TN (true negatives) represents the number of all possible PPIs that appear in neither the testing dataset nor the benchmark dataset; and FP (false positives) represents the number of PPIs in testing dataset but are not in the benchmark dataset. The Jaccard coefficient, recall, and precision of the benchmark and testing datasets considered in this work are given in Figures 1, 7, 8, 9, and 10.

### STRING score distribution of “overlap PPI number ratio”

In order to find which STRING score region has a higher percentage of overlapping PPIs with the B2H PPI dataset, STRING score distribution of “overlap PPI number ratio” between the STRING predicted functional associations dataset and the *M. tuberculosis* H37Rv B2H PPI dataset were calculated and plotted in Figure 2. At each score interval of 10, the “overlap PPI number ratio” is defined as the number of overlapping PPIs divided by the total number of PPIs in that interval. For example, if there are 300 PPIs from the STRING predicted functional associations dataset are in score range 150~160, and among these 300 PPIs there are 30 PPIs overlapping with the B2H PPI dataset, then in this score range 150~160 the “overlap PPI number ratio” is 30/300 = 0.1. We calculate all the “overlap PPI number ratio” in each interval, STRING score ranging from 150 to 1000, and the distribution of the ratios are plotted in Figure 2.

### PPI datasets analysis by PPI functional intensity matrix

COG function annotations are used in the analysis. We calculate the percentage of PPIs in each cell in the matrix. In the matrix, each cell is a combination of the COG functional category between a pair of proteins. The percentage (“intensity”) of PPIs in each cell is the number of PPIs in that cell divided by the total number of PPIs in the dataset. The number of PPIs in a certain cell means the number of PPIs fit the functional categories combination; that is, one partner has been annotated with the COG category on the x axis, the other partner has been annotated with the COG category on the y axis. If “m” PPIs have “J-K” annotations in a dataset that has “n” PPIs—and “J-K” annotations means one partner form J COG functional category, the other partner from “K” COG functional category—then the PPI functional intensity in the “J-K” cell is “m/n”. This is slightly different from the conventional approach of calculating the PPI functional intensity matrix; but it is good enough to convey broad information on the functional distribution of the PPI datasets tested. The PPI functional intensity matrices generated in this work are displayed in Figure 3.

### GO term annotation, informative GO term identification and PPI datasets assessments

*M. tuberculosis* H37Rv, *C. jejuni* NCTC11168 and *Synechocystis* sp. PCC6803 proteins are annotated with GO terms using InterProScan [25]. GO terms are organized into three separate hierarchical ontologies—viz., cellular component terms (CC), molecular function terms (MF), and biological process terms (BP). A protein that is annotated by a particular GO term is considered to be annotated by all ancestor terms (in the corresponding hierarchical ontology) of that GO term—that is, the so-called “through-path” rule is applied. As top-level GO terms tend to be annotated to many proteins and leaf-level GO terms to very few proteins, in order to avoid bias in our analysis, we keep only “informative” GO terms for analysis. An informative GO term is defined as a GO term that has at least 30 proteins assigned to it or its descendants and none of its child terms have 30 or more proteins assigned to it. This way, exactly one GO term is considered in any through path. Moreover, each GO term considered is at the finest resolution possible while being annotated to a sufficiently large number of proteins (≥ 30) for a valid analysis.

A pair of proteins come into contact with each other and interacts to perform a function. If the GO term annotations of the proteins in an organism are complete, we can expect such a pair of interacting proteins to have at least one informative GO term annotation in common. Therefore, a predicted or reported PPI is more likely to be a false positive when the two proteins in the PPI do not have an informative GO term annotated to them in common. We can therefore gauge the quality of a PPI dataset by calculating the percentage of PPIs in the dataset (where both proteins in the PPI have informative GO term annotations) that has “coherent” informative GO term annotations. A PPI is said to have coherent informative GO term annotation if the two proteins in the PPI have an informative GO term annotation in common.

However, the percentage of PPIs in a dataset that have coherent informative GO terms can be affected by the number of informative GO terms and by biases in the distribution of proteins these informative GO terms are annotated to. For example, if only one informative GO term was available in the organism, then 100% of the annotated PPIs would be coherent. Thus, to better assess the quality of a PPI dataset by coherence of informative GO term annotations, we need to compare the percentage of coherently annotated PPIs in the dataset to appropriately generate random PPI datasets. In particular, a high ratio (named “Info GO ratio”) of the percentage of coherently annotated PPIs in the PPI dataset compared to that of the random PPI dataset suggests that PPI dataset is likely to be of high quality. We generate random PPI network using the Random Network Plug-in [26] in Cytoscape [27]. The percentage of PPIs that have coherent informative GO term annotations in the PPI datasets considered in this work is given in Figures 4 and 6, as well as Tables 2 and 8.

### Gene expression profile correlation

A pair of proteins needs to be expressed in the same location and at the same time in order to interact. Thus the highly correlated gene expression profiles of two genes suggest the likely interaction of their two gene products. Genome-wide gene expression profiles under metabolic inhibitory and control conditions [1] are used here to gauge the correlation of gene expression profiles. Given any protein pair (x, y), the correlation of the expression profiles (G_x_, G_y_) of the two underlying genes are calculated by Pearson correlation coefficient as follows[28],

Here, n is the number of conditions for the expression profiles; x_i_ is the i^th^ expression value of the gene corresponding to protein x in the gene expression profiles G_x_; and  is the average value of G_x_.

The distributions of Pearson correlation values for PPIs in the datasets considered here are plotted in Figure 5.

### *M. tuberculosis* H37Rv interolog prediction

An interolog is a conserved interaction between a pair of proteins which have interacting homologs in another organism. We use two different methods to identify homologs between *M. tuberculosis* H37Rv and the other 629 organisms in STRING. In the first method, we use Inparanoid [29] with default parameter setting. In the second method, we use BLASTP [30] with HVAL > 20 and E-VALUE < 1*e-6. In the third method, we use BLASTP with PIDE > 30, length of the alignment > 20% of query sequence length, and E-VALUE < 1*e-10.

Here, the HVAL [31] is the percentage identities threshold based on the HSSP curve. It is defined as:

where PIDE is the percentage identities of the BLASTP hit and L is the length of the alignment.

### Analysis of characteristics of PPI datasets using integrated pathway gene pair relationships

In order to better understand what the predicted PPIs in STRING really correspond to, we use the pair-wise relationships of proteins/genes from the XML files of each of the pathways in KEGG [12], WikiPathways [13,14] and BioCyc [15]. The XML files in these three major pathway databases follow different formats. KEGG pathways information is stored in KGML format. WikiPathways [13,14] pathway information is recorded in GPML format. BioCyc (specifically, MTBRVcyc) pathway information is stored in BioPAX format [32]. Mining and retrieving pair-wise protein/gene relationships from KGML and GPML are mainly by using our in-house program. For BioPAX [32], we convert BioPAX files into simple SIF files use paxtools [32]; then we make some node mapping to retrieve the protein/gene pair relationships. These gene pair relationships are classified in accordance to KEGG [12] into four categories: ECrel, PPrel, GErel, PCrel; see Table 7. In particular, ECrel comprises protein/gene pairs that catalyze/form adjacent reaction steps in enzymatic pathways, and PPrel comprises protein-protein interactions such as binding and modifications.

### Software packages, program tools, materials and datasets

The software packages and program tools used in this study are:

(a) Protein Sequence Alignment software package: BLASTP [30,33].

(b) Orthologues Identification software package:

Inparanoid stand-alone package, Version 4.1 [29].

(c) BioPAX pathway file processing software package:

Paxtools [32].

(d) Random PPI network generation:

Random Network Plug-in [26] for Cytoscape [27].

(e) Protein Signature annotation program:

InterProScan [25].

The datasets used in this study are:

(a) *M. tuberculosis* H37Rv Gene Expression Datasets [1].

(b) Pathways Datasets:

KEGG [12].

WikiPathways [13].

BioCyc [15].

(c) PPI datasets:

*M. tuberculosis* H37Rv high-throughput B2H PPI dataset [7].

*M. tuberculosis* H37Rv in STRING [3].

*S. cerevisiae* protein complexes dataset obtained from Wodak Lab [11].

*S. cerevisiae* two-hybrid PPI dataset [10].

*C. jejuni* two-hybrid PPI dataset [8].

*Synechocystis* two-hybrid PPI dataset [9].

(d) *M. tuberculosis* H37Rv proteins function annotations:

COG functional categories annotation [34].

(e) *S. cerevisiae* GO annotations downloaded from Gene Ontology website [35] dated 6/12/2010.

## Competing interests

The authors declare that they have no competing interests.

## Authors' contributions

This work was jointly conceived, planned, analyzed, and written up by LW and HZ. The analytical experiments were performed by HZ.

## Supplementary Material

Additional file 1 **Reliable *M. tuberculosis* H37Rv B2H PPI datasets**. Description: the reliable *M. tuberculosis* H37Rv B2H PPI datasets, list in four text files, tab delimited. Click here for file
